# Youth's (Un)willingness to work in agriculture sector

**DOI:** 10.3389/fpubh.2022.937657

**Published:** 2022-08-29

**Authors:** Laura Girdziute, Erika Besuspariene, Ausra Nausediene, Anastasija Novikova, Jarkko Leppala, Martina Jakob

**Affiliations:** ^1^Vytautas Magnus University, Kaunas, Lithuania; ^2^Natural Resources Institute Finland, Helsinki, Finland; ^3^Leibniz Institute for Agricultural Engineering and Bioeconomy e.V., Potsdam, Germany

**Keywords:** agriculture, perceptions, youth, willingness to work, work in agriculture

## Abstract

Work in agriculture is a significant area of research that highlights the problem of the integration of young people in the former, in particular, in the recent period. Work in agriculture is hard and not prestigious, and young people tend to leave rural areas in the search for alternative activities in urban areas. The study addresses the problem of how the youth should be integrated into agricultural workforce by focusing on identification of the reasons behind the (un)willingness to work in agriculture. The aim of the study is to assess the reasons behind the youth's (un)willingness of work in agriculture, using Lithuania as the case study. The data were collected by means of a questionnaire designed to investigate the perception and opinions toward work in agriculture. The Binary Logistic Regression was used to identify the factors affecting the youth's opinion about (un)willingness to work in agriculture. The study analyzed 430 young people 's responses to the questionnaires survey. The BLR has revealed that youth's unwillingness to work in agriculture is mostly affected by gender, area of residence and youths' beliefs that work in agriculture does not provide any opportunities for self-realization. In summary, this paper argues that the major motivation to work in agriculture is associated with having parents who are engaged in agricultural activities, love of animals and natural environment, and the availability of specialized training. The findings have confirmed the need to attract young people to work in agriculture. Its results are necessary for the scientific community, policy makers, farmers, and practitioners exploring the possibilities for integration of the youth into the agricultural workforce.

## Introduction

Agriculture requires a growing workforce in order to meet the increasing demand for products. Unfortunately, employment has been declining dramatically in agriculture (to just 26.76% of the workforce in 2019 worldwide, according to the data by the World Bank^1^). Moreover, work in agriculture may also affect people's health and quality of social life. Agriculture is also the sector with the highest risk indexes (1), and can be described as a specific business that is hazardous due to the chemicals used (2), requiring hard work, such as heavy physical activity, frequently unconventional working hours, etc. (3). Significant decisions that influence an individual and society's destinies place particular focus on the youth (4). In today's world, the youth have an important role as the future of food security and sustainable agriculture depend on them. Older farmers are generally considered to be used to conventional farming traditions and methods, and they will arguably be reluctant to adopt new technologies or innovations in their farms (5).

To address the above-mentioned problem, the EU have already been running several programmes to encourage young people to take up farming. For example, the European Common Agricultural Policy (CAP) programmes help farmers under 40 set up a business; member states offer young farmers an up to 25% bonus on top of their CAP subsidies, and advisory services and training schemes have been offered under the rural development programmes. Despite the above measures, the number of young farmers in the agriculture sector is still very small in the EU. Attraction of the youth to agriculture sector becomes more important now that the Green Deal guidelines have been announced. To attract young people to work in agriculture, it is necessary to make agriculture more dynamic and appealing compared to its present state. Moreover, young people need to be persuaded to view the sector more positively than they do now (6). The recent research also highlights that the youth remain an important component of the agricultural labor force (7, 8) in the aspiration to upgrade agricultural practices and utilize new technologies for greater agricultural growth. Youth involvement drives labor diversification trends from predominantly rural agricultural activities to more urban focused manufacturing and service sector activities.

Meanwhile, the youth tend to migrate from rural to urban areas (9, 10), and the agriculture sector is naturally facing the challenge of unemployment. Furthermore, high levels of unemployment lead young people out of rural areas (11). Youth unemployment in agriculture is one of the crucial questions related to development of agriculture sector, rural areas, communities, etc. (12, 13).

The “young farmer problem” articulates the issue of aging of farmer population (14). Farmers are getting older all around the world, and this aggravates the aging problem, which has become one of the key issues in agriculture sector (14). Only 5.6% of the European farms were managed by farmers under the age of 35, while over 31% of the farmers were over the age of 65 (15), and just 10.6% of the farms had managers under the age of 40 in 2016 (Eurostat^2^). The data correlate with Kołodziejczak (16) research findings, which have shown that one of the highest rates of employment in agriculture were registered in Romania, Bulgaria, Poland, Latvia, Lithuania and Croatia, while the rate of young people involved in the agricultural workforce was insignificant. The same inconsistencies were found by Janeska (17) analyzing the employment in the Republic of North Macedonia, showing an intensified process of demographic aging in agriculture. The situation of aging work force in the agriculture sector is not specific to the EU only, and, according to Jöhr (18), the average age of farmers in the US was then 58, in Japan – 67.

Agriculture creates negative stereotypes, and young people are moving from rural to urban areas. Still a major problem in the EU is the high level of youth unemployment (under 25), which was 17% in 2021(Eurostat^3^). Young people's attitudes toward work, in agriculture are formed at an early age (19), so it is important to consider the reasons behind the encouragement of a certain job choice. Negative stereotypes do not support EU agriculture in shifting to a sustainable future. That highlights how important it is to attract young people to agriculture. A comprehensive literature review has revealed the existence of certain stereotypes relating to the farmers' image, as well as working conditions, social status and other (3, 20–22). Lundy et al. (23) found out that both young people and adults had a stereotyped concept of agricultural workers. The workers were associated with a rugged, tan man working outside. Therefore, despite the multiple innovations being implemented by the agriculture as a job creator to reduce unemployment in the EU, attracting young people remains a challenge. To build a different image of agriculture among the youth, it is necessary to change young people's mindset today, but this requires assessment of how young people view agriculture at present. In this context, the present study focuses on analysis of the factors affecting the youth's (un)willingness to work in agriculture using Lithuania as a case study. This analysis may allow and its results are necessary for the scientific community, policy makers, farmers, and practitioners exploring the possibilities for integration of the youth into the agricultural workforce.

## Materials and methods

### Case study: Labor situation in agriculture in Lithuania

Lithuania is characterized by a strong focus on the development of rural regions, as approximately one-third of Lithuania's population live in the rural areas.

According to the National Land Service^4^ in Lithuania, more than 80% of the area are rural, 52 % of the surface land is agricultural land, and arable land covers 46% in 2021. Therefore, Lithuania has a strong potential for work in the agricultural sector. According to the year 2019 database of the Lithuanian Employment Service^5^, the role of the agriculture sector in the Lithuanian economy was declining over the last decade, and the value added of agriculture declined as well. This was influenced by the decline of rural population: the average of 9 thousand people would leave rural areas for urban areas every year. According to the population migration data, the rural population halved within the last 50-year period. According to the data by Statistics Lithuania^6^, the number of permanent residents in rural areas in Lithuania decreased by 26.5 thousand between 2016 and 2019. The decreasing population in rural areas is affected not only by urbanization, but also by the growing size of farms. The average farm size by agricultural area increased from 11 hectare to 20 hectares from 2005 to 2016 (Statistics Lithuania). The implications of the growing size of farms influence the integration of young people into agriculture with the possibility to have an own farm. However, farm growth cannot be viewed only as a negative phenomenon as it creates new workplaces. The number of employees in agriculture increased almost twice over the decades, from 24 k employees to 40 k employees between 2005 and 2016 (Statistics Lithuania). Agriculture remains an important employer in Lithuania and contributes to the development of the country's economy, employment of the population, food supply, etc.

The Lithuanian agriculture sector has been undergoing substantial restructuring upon Lithuania's accession to the EU in 2004 (24, 25). Main changes have been prompted by adaptation of the EU CAP (24), the economic crisis in 2009, the Russian embargo in 2012 (26), as well as the recent Covid-19 pandemic. The Lithuanian government has been focusing on various instruments to support the fintech and service sectors, and the average salary in agriculture has decreased compared to the national average salary (26). The threat posed to agriculture by the rising wages in other sectors is also mentioned in the report by the Lithuanian Institute of Agrarian Economics (27) on the assessment of the economic, social and environmental situation of Lithuanian agriculture and rural areas. The above facts may be considered as some of the reasons behind young people's reluctance to choose to work in agriculture. Agriculture is becoming unattractive due to lower wages, economic and political difficulties, and governmental support to other business areas that also need specialists. These factors may lead to the situation where rural youth opt for non-agricultural careers and move to the cities.

Despite the above implications, the agriculture sector is one of the core economic sectors in Lithuania and employs about 8% of the national labor force (26). The analysis of statistical data shows that Lithuania has a rural population aging problem, as farmers are predominantly in the age group above 50. A very small share of those employed in agriculture are under the age of 29 (Statistics Lithuania). According to the data by Statistics Lithuania, the internal migration of the population from rural to urban areas in 2020 increased by as many as 14% compared to 2016. According to the data by the Lithuanian Employment Service^7^, persons over the age of 50 made the largest share of seasonal employees in agriculture, making about 46% of all the employed in 2020. The report by the Lithuanian Institute of Agrarian Economics (27) points at the issue of aging of the agrarian society in Lithuania that leads to increased reluctance among the young people to live and work in the countryside. This is partly due to the unattractiveness of farming as an economic activity (its routine nature, uninterrupted production cycle, unconventional working days). There has been an increase in the number of young farmers, but they are not actively engaged in farming activities despite enjoying the benefits of residence in rural areas. This poses a threat to the uninterrupted generational change in agriculture and the continuation of farming traditions.

In 2020, youth (up to 24 years) unemployment accounted for 11.9% of the unemployed in Lithuania, while unemployment in other age groups ranged from 5 to 8% (Statistics Lithuania). The number of people employed in agriculture could actually be higher taking in account the lack of workforce in the agriculture sector and the high unemployment rate among young people. According to the Lithuanian Employment Service^8^, the agriculture sector is facing a labor shortage of 27.3% in 2021. One of the negative factors is that the agriculture sector is facing the deficit of educated labor force in Lithuania. Another limiting factor is that rural regions are historically associated with agriculture in Lithuania, creating barriers to change the economic structure of regions in Lithuania (25).

Dairy and animal husbandry activities prevailed in Lithuania before its accession to the EU. Afterwards, a lot of farms started growing grain and rapeseed crops, today accounting for about 55%, which has led to the need for structural changes in the agriculture of rural regions (25). This issue is caused by the lack of innovative development of higher value-added products and shortage of labor, in particular skilled labor, in rural areas (27). Volkov et al. (24) have disclosed another existing issue related to large number of farms on the Lithuanian market despite the growth in farm size. Certain farms have low profitability, and given that the majority of farms are run by older people approaching retirement, it is difficult to maintain the previous level of labor demand in agriculture. This reveals the existing issue of a high unemployment rate among the Lithuanian youth and shortage of labor force in agriculture. This shows that the issues of youth employment in agriculture needs to be discussed and addressed.

In Lithuania, same as in the EU, it is important to take into account the recommendation by the European Council (2020/C 372/01) (28) “A Bridge to Jobs – Reinforcing the Youth Guarantee” superseding the 2013 Youth Guarantee. The recommendations include strengthening of the prevention of youth unemployment and inactivity; preparation of young people for the new labor market; introduction of the youth to the need for lifelong learning; in-service training or retraining with a focus on 'green' or digital skills, etc. Therefore, the present study may help reveal the reasons behind the young people's unwillingness to work in agriculture, and the results of the study may contribute to an informed decision-making related to reduction of the youth unemployment. The report by the Lithuanian Institute of Agrarian Economics (27) on the assessment of the economic, social and environmental situation of Lithuanian agriculture and rural areas has revealed a series of existing related issues: young farmers' reluctance to pursue development in regions remote from major cities; lack of free land; lack of capital for young farmers to set up new farms; deteriorating infrastructure in rural areas as a barrier to generational change; rising wages in other sectors of the economy affecting the attractiveness of farming for young people.

In order to address young people's reluctance to live in rural areas and work in agriculture, it is necessary to put a stop to the deterioration of the demographic situation in rural areas and redirect the population migration in Lithuania. In order to keep young people in the countryside and attract new ones, conditions must be created for them to engage in the desired activities in rural areas, enabling them to generate sufficient income. Therefore, favorable conditions must be created for business start-up initiatives in rural areas (27).

### Research methodology

Youth's (un)willingness to work in agriculture sector was measured by using two binary variables, which took a value of 1 when an individual i) had the priority to work in the agricultural sector (PRIOR_AGR); and ii) was willing to work in the agricultural sector (LIKE_AGR), and 0 – otherwise. Given that the dependent variable was dichotomous, its relationship with independent (explanatory) variables was estimated using a binary logistic regression model, as in (29, 30). Equation 1 shows the general formula for BLR model:


$$Y_{ij}=\alpha_{i}+\beta_{j}\sum_{ix1}^{n}X_{ij}+e_{i}$$

Where: *Y*_*ij*_ is the dependent variable (priority to work in the agricultural sector or willingness to work in the agricultural sector), $\overset{n}{\underset{jx1}{\sum }}X_{ij}$ is the sum of independent (explanatory) variables (socio-demographical characteristics and perception variables) for *j*th of the respondent, α_*j*_ … β_*j*_, are the estimated coefficients, *e*_*i*_−the error term.

The study considered the following socio-demographical characteristics: gender (encoded as 1=male, 0 = female), area of residence (RESID) (encoded as 1 = village, 0 = city), family persons engaged in agricultural activities (PER_AGR), and relatives or acquaintances working in agriculture (REL_AGR) (encoded as 1 = yes, 0 = no). In addition, three groups of questions revealing perceptions, which could encourage/discourage people to work in agriculture, were analyzed: i) individual perceptions; ii) economic perceptions; and iii) social perceptions, as proposed by Magagula et al. (31). The answers to the questions, such as what conditions would encourage employment in the agriculture sector (in the groups of perceptions), were ranked based on Likert scale (1 = totally disagree; 2 = disagree; 3 = do not have an opinion; 4 = agree; 5 = totally agree). Explanation of perceptions and statement variables presented in Table 1. Statements in the questionnaire were constructed taking into account/under the assumption that the stereotypes relating to the farmers' image and work in agriculture do exist. Here, mostly based on (6b) and other literature, questions were developed to help the researchers identify the aspects of different stereotypes that are relevant to the youth. Later the developed questionnaire was discussed and approved in the group meetings of the COST Action.

**Table 1:** Description of perceptions independent variables used in the BLR models.

**Perceptions**	**Statement in the questionnaire**
Individual	• Young people should work in agriculture (AGR_YOU) • It is an opportunity to have your own farm (FARM_OP) • I would like to work in agriculture because I love nature and animals (LIKE_NAT) • I would choose to work in agriculture only abroad (AGR_ABR) • No conditions would encourage me to work (WORK_NO) • Specialized training in agriculture would encourage me to work (SPEC_TR)
Economic	• Work in agriculture is low paid (AGR_PAID) • Agricultural work is seasonal (AGR_SEA) • Working in agriculture does not provide opportunities for self-realization (AGR_SR) • Working in agriculture is profitable (AGR_PROF) • The agricultural sector is well developed and will always remain significant (AGR_SIG) • No development in agriculture (NO_DEV) • Modern farmers have a lot of financial resources (FIN_RES) • Technological innovations make work in agriculture more attractive (AGR_TECH) • Higher salary / wage (WAGE)
Social	• Work in agriculture is dirty (AGR_DIRT) • Working in agriculture is physically difficult (AGR_DIF) • Working in agriculture is dangerous (AGR_DAN) • Working in agriculture is not prestigious (AGR_NOPR) • Working in a natural environment (WORK_ENW) • Agricultural work is for unskilled workers (AGR_UNSK) • Working in agriculture is a life-style (AGR_LS) • Work in agriculture is responsible (AGR_RES) • Incomplete social life in rural areas for young people (AGR_NOSL) • Flexible work schedule (WORK_SCHED) • Nature of work (WORK_NAT) • Feeling the difference between urban and rural lifestyles (FEEL_DIF)

In order to investigate whether work in agriculture would be a priority for young people (PRIOR_AGR) and whether they would like to work in agriculture (LIKE_AGR), two BLR models were created. Model 1 was based on the question *would working in the agricultural sector be a priority for you*, while model 2 was based on the question *would you like to work in the agricultural sector*.

The models were developed using the Stepwise Method of Forward Stepwise function in SPSS to avoid the problem of multicollinearity. Independent variables, tested as potential predictor variables, were socio-demographic characteristics: gender (GENDER), area of residence (RESID), family persons engaged in agricultural activities (PER_AGR), and relatives or acquaintances working in agriculture (REL_AGR). They were coded as dummy variables value (1 and 0). Other independent variables of individual, economic and social perceptions, coded to Likert scale, had the values of 1 to 5. In total, 31 (27 perception variables, and 4 socio-demographic variables) factors were included in each model and checked. All statistically insignificant variables were excluded from the final BLR models, i.e., final BLR models were considered and the results were analyzed using only the significant variables.

The target population of research are young people in Lithuania. According to the definition of youth population provided by the European Commission (32), this is the total number of young people in the age groups 15–19, 20–24, and 25–29 living in a member state of the European Union on January 1st. The target population of the research was young people in the age group 15–19, since they had not yet chosen the prospective profession, were not studying a particular subject at university, college or vocational school. This choice was based on the aim to explore the factors behind the young people's willingness to work in agriculture, as they had probably not yet chosen a field of study or direction of work opportunities and still had the possibility to plan their occupation or even studies in agriculture field. Therefore, the focus was placed on young people, still learning at school.

As the target population was selected young people in Lithuania we considered Lithuanian law principles that young people till 13 years old need to have permission of the parents to be asked. Here, according to Civil Code of the Republic of Lithuania^9^ article 2.8 youth from 14 till 18 years could make decisions and small/ daily contracts, receive revenue from activities and control their finance and banks. In other words, these group of young people takes responsibilities themselves for obligations, and could be suitable to participate in present research about their perceptions toward working in agriculture.

### Sampling characteristics

The main survey was conducted in the period from December 2020 to April 2021. Due to the pandemic situation related to Covid-19, the survey was implemented online in the attempt to involve young people willing to fill out the questionnaire. Therefore, 444 questionnaires were completed, with only a few questionnaires rejected as completed inappropriately. Data of 430 questionnaires were registered and analyzed. According to Israel (33) and in view of the target population size, the number of questionnaires collected ensure a statistical error between 5 and 7%. Hence, sample size *N* = 430 assured that the analysis of 430 questionnaires would reflect the total sample (*N* = 130 926; young people the age 15–19 living in Lithuania in 2020 by Eurostat), with 95 % confidence level and ± 5% sampling error. The descriptive statistics of the respondents' main sociodemographic characteristics are presented in Table 2.

**Table 2:** Respondents' socio-demographic profile (*N* = 430).

**Variables**	**Study sample**	
	**N**	**%**
**Gender**		
Male	166	38.6
Female	264	61.4
**Age (15-19)**		
15–16	144	66.66
17–19	286	33.4
**Area of residence**		
City	274	63.7
Village	156	36.3

Identifying the factors of youth's motivation to work in agriculture toward their individual, economic and social perceptions were analyzed. Descriptive statistics have suggested that higher salary could seem to be the best motivating factor to choose work in agriculture, and more than 73% of the respondents agreed with this statement. It was followed by the flexible work schedule (66%). About half of the respondents agreed that the nature of the work and conditions could encourage them to work for the agricultural sector. A few respondents would be motivated to work in agriculture if offered various training and qualification courses. It turned out that only about 15% of the respondents would not work in agriculture under any condition. Half of the respondents thought that the agricultural sector would remain significant and was well-developed, while the other respondents did not share that opinion and believed that there was no development in the agriculture sector. Only 20% of the respondents stated that the social life was not fulfilling for young people engaged in agriculture. More than 60% of the respondents thought that the technological innovations transformed agriculture into an attractive place to work.

The major share of the respondents thought that work in agriculture required a great deal of responsibility. A considerable part of the respondents did not agree that work in agriculture was for unskilled people. More than half of the respondents had no opinion about self-realization in the working in agriculture. It should be noted that the majority of them had difficulties in finding work in agriculture, and the minority thought that work in agriculture was low paid. About half of the respondents had no opinion about the attractiveness of work in agriculture. The results of the survey showed that about half of the respondents did not have any opinion on whether young people should work in agriculture or not. Most of them agreed that work in agriculture provided an opportunity to have own business, and more than half of the respondents disagreed to the statement claiming that they would choose to work in agriculture abroad only.

## Results

Using Forward Stepwise method, 10- and 6-step actions were performed in the first and in the second model respectively. The selected models were formed in step 6 of both models. Table 3 provides information on the goodness of fit of BLR model.

**Table 3:** Information of the goodness of model fit.

**Model**	**Omnibus tests of model's coefficients**	**Chi-Square**	**df**	**Sig**.
1	Step	4.853	1	0.028
	Block	88.424	6	0.000
	Model	88.424	6	0.000
2	Step	6.388	1	0.011
	Block	131.045	6	0.000
	Model	131.045	6	0.000

As presented in Table 3, the Omnibus tests assess the goodness of fit of the models incorporating the statistically significant variables. Sig. (*p* = 0.000) shows that model is statistically significant and the dependent variable is well predicted. The Chi-Square test explained a significant amount of the original variability, *x*^2^ (6, *N* = 430) = 88.424 in the first model and *x*^2^ (6, *N* = 430) = 131.045 in the second model. The data of models indicate that both models are reliable; therefore, the obtained models' results can be interpreted and the existing stereotypes affecting the Lithuanian young people's desire to work in agriculture can be assessed. Model 1 presents the cases where young people see the priority in working in agriculture (see Table 4).

**Table 4:** Result of BLR model 1.

	**B**	**S.E**.	**Wald**	**df**	**sig**	**Exp(B)**	**B**
GENDER	−0.634	0.288	4.853	1	0.028	0.530	−0.634
RESID	−0.915	0.290	9.928	1	0.002	0.401	−0.915
PER_AGR	0.936	0.292	10.297	1	0.001	2.549	0.936
LIKE_NAT	0.686	0.172	15.901	1	0.000	1.986	0.686
AGR_YOU	0.496	0.165	9.100	1	0.003	1.643	0.496
AGR_NOSL	−0.381	0.144	7.005	1	0.008	0.683	−0.381
Constant	−4.103	0.920	19.907	1	0.000	0.017	−4.103
−2 Log likelihood	321.787						
**Pseudo R-squared:**							
Cox and Snell	0.186						
Nagelkerke	0.302						
Overall Percentage	84.20%						
Observations	430						

Variable significant at 1%.

Table 4 shows the results of the BLR model 1 and its goodness of fit. The Nagelkerke modification is considered to be a more reliable measure of the ratio compared to Cox and Snell's. In this model, Nagelkerke, *R*^2^ accounts for 0.302, indicating 30.2 % relationship between the predictors and the prediction. The overall percentage of correct recognition of the first model is 84.20 %. The BLR results show that socio-demographical characteristics, individual, and social perceptions about the work in agriculture influenced youth' decision to see the work in agriculture as priority: i) women were less likely to choose work in agriculture; ii) the young people living in urban areas were less willing to choose agriculture as the priority in comparison to those living in rural areas. iii) youth whose parents were engaged in agricultural activities iv) and who loved nature and animals (LIKE_NAT), were most likely to choose the work in agriculture as a priority. The respondents who believed that young people should work in agriculture (AGR_YOU) were more inclined to choose work in agriculture as a priority. However, the opinion related to unsatisfactory social life in rural areas (AGR_NOSL) for young people was associated with decreased priority among the young people to work in agriculture.

Table 5 shows the results of the BLR model 2 and its goodness of fit. The R-squared values. Here, Nagelkerke, *R*^2^ accounts for 0.399, indicating a 39.9 % relationship between the predictors and the prediction. The overall percentage of correct recognition of the second model is 83.3%, indicating that the both models provide correct classification of the cases.

**Table 5:** Result of BLR model 2.

	**B**	**S.E**.	**Wald**	**df**	**sig**	**Exp(B)**	**B**
GENDER	−1.221	0.296	17.076	1	0.000	0.295	−1.221
SPEC_TR	0.394	0.154	6.575	1	0.010	1.483	0.394
LIKE_NAT	1.013	0.185	29.875	1	0.000	2.753	1.013
AGR_SR	−0.659	0.172	14.639	1	0.000	0.517	−0.659
AGR_PROF	0.457	0.184	6.141	1	0.013	1.580	0.457
AGR_YOU	0.528	0.165	10.294	1	0.001	1.696	0.528
Constant	−7.312	1.183	38.214	1	0.000	0.001	−7.312
−2 Log likelihood	330.547						
**Pseudo R-squared:**							
Cox and Snell	0.263						
Nagelkerke	0.399						
Overall Percentage	83.30%						
Observations	430						

Variable significant at 1%.

Table 5 shows the results of BLR analysis, presenting youth's willingness to work in agriculture sector. The BLR results show that socio-demographical characteristics, individual, economic and social perceptions about the work in agriculture variable influence youth's willingness to work in agriculture. The results show that gender had a negative effect and significantly reduced the willingness to work in agriculture (as in BLR model 1 as well), and women were less likely willing to work in agriculture (by 1.22 times) than men. The findings have shown that youth's willingness to work in agriculture increased due to specialized training in agriculture (SPEC_TR), love of nature and animals (LIKE_NAT), and as well as youth's belief that they should work in agriculture (AGR_YOU) and opinion that work in agriculture was profitable (AGR_PROF). Youth's belief that work in agriculture did not offer any opportunities for self-realization (AGR_SR) reduced their willingness to work in agriculture. It could be assumed that the research showed the importance of the financial factors when choosing to work in agriculture.

## Discussion

The findings of the present research about youth's (un)willingness to work in agriculture and the factors determining this decision are in line with previous studies. The results have suggested that gender was the factor influencing the willingness to work in agriculture. Similar results were found by Elias et al. (34), namely, that females did not seek a career in agriculture dominated by males. Recent research in Lithuania has shown that there is no large gender gap in agriculture, nonetheless it also indicates that women are more interested in non-agricultural activities, which coincides with our research (34).

The findings show that the area of residence (youth from rural areas were more willing to choose work in agriculture as a priority) had a statistically significant influence on prioritization of work in agriculture. This is an expected result supported by the literature (35), revealing that the youth who has strong connection with their home village will likely stay to work at their family farm. Otherwise, the youth is usually willing to migrate because of family reasons, job opportunities or education (35). Ridha and Wahyu (36) and Aziz and Naem (37) found that the motivation to work in the agriculture sector would stem from parents, family, who are working in agriculture. The research by Simõesa and do Rio (38) revealed that positive perceptions about the work in agriculture sector originating in the family increased the youth's motivation to work in this sector. The same findings were indicated by the present research. Our research has revealed that loving nature and animals increased youth's willingness to choose work in agriculture, and seeing it as priority, thus substantiating the conclusions of the previous research (39).

Our results underlined that specialized training in agriculture (SPEC_TR) had a statistically significant impact on the youth's intentions to work in agriculture. This implies that having a specialized training would also motivate young people to work in agriculture, thus substantiating the findings by Cecchini et al. (1), Magagula et al. (29), and Simõesa and do Rio (38). Present research found a positive link between positive attitude and intention/willingness to work in agriculture, which is in line with the previous studies (40, 41). However, youth's negative opinion about opportunities for self-realization (AGR_SR) in agriculture reduced their intentions to work in agriculture. Similar results were found by Akrong and Kotu (42) stressing the negative perceptions among the youth about agribusiness. Vankov et al. (43) have noticed that the youth's desire of self-realization and desire to become entrepreneur may be affected by cultural or geographical factors. In particular, the authors draw attention to the fact that in the developed nations, the youth have lower intentions than those in the developing countries. Meanwhile, Zhartay et al. (44) mark specific age, personal characteristics of young people, their social status, mobility, activity and adaptability as important factors for self-realization in entrepreneurial. Therefore, decision to start business in the agriculture sector may be related less to the specifics of agriculture sector and more with the social-demographic reasons.

This was an expected result as work in agriculture was not economically encouraging and required wider promotion among the young people (31, 36, 45, 46). According to Finger and Benni (47) farm income depends on such variables as the increasing complexity of farms, increasing risk of exposure, and increasing complexity of agricultural policies and policy measures. It is therefore understandable why this sector does not look so attractive for the youth.

The present study is in line with the CFS Policy Recommendations on Promoting Youth Engagement and Employment in Agriculture and Food Systems (48) stressing the same aspects as equity and distribution of resources across generations, appropriate infrastructure for young people life, and ensuring appropriate social life. The present study showed the need to attract young people to work in agriculture and the need for policy instruments. This finding supports the results of Mujčinović et al. (49) who analyzed the possibility to tackle the need for the youth by the means of agricultural and rural development policy in 28 EU countries and Bosnia and Herzegovina, North Macedonia and Serbia. Their comparative analysis indicated the need for the policy aimed at attracting young people to work in agriculture. However, they also found differences between the regional development policies among post-transitional countries and between the potential to adapt modern European practices and policies.

## Conclusions

The present study was a part of the COST action CA16123 “Safety Culture and Risk Management in Agriculture.” The focus of the study was to analyze the reasons behind the youth's (un)willingness of work in agriculture, using Lithuania as the case study. Ac-cording to the agricultural characteristics of Lithuania, the agriculture sector plays an important role for the whole economy of the country. The obtained research results may find practical application in development of programs for attraction and integration of the youth as the agricultural workforce. Agriculture is developing, innovating, and requires skilled workers. Therefore, agriculture should be presented as an innovative, technologically advanced sector, where self-realization is possible by creating own business. Only in this way young people will be attracted to agriculture and accelerate its efficiency, sustainability, contributing to achievement of the goals of the Green Deal. The necessity to develop infrastructure and create attractive leisure facilities for young people in rural are-as is another important issue in terms of youth attraction in agriculture. Funding for agriculture must be properly targeted at the infrastructure necessary for young people's social life and application of new technologies in agriculture.

Summarizing the results of current research, it can be stated that there are a few positive stereotypes: i) the youth see the need of trainings for working in agriculture; ii) they think that young people should work in agriculture. However, the research has revealed that the Lithuanian youth pointed at a number of negative stereotypes influencing their decision regarding the choice to work in agriculture: i) the youth thought that the social life in rural areas for young people was incomplete, and ii) they believed that there was no opportunity for self-realization in agricultural work. In addition, it should be noted that these stereotypes could be adjusted using a variety of measures, such as training, educational programs, etc. This could help change youth perceptions toward work in agriculture, taking into consideration their personal characteristics. Our research has revealed that one of the most important personal qualities in young people is love of nature and animals, making them willing to work in agriculture.

In addition to the positive and negative stereotypes about agriculture, other important socio-demographic characteristics were noticed. The desire to work in agriculture was determined by family activities in this field, as well as the living area. This only confirms that the formation of positive and negative stereotypes in agriculture can be influenced by the place of residence (city or village) and family activities (work in agriculture or another sector). Another important socio-demographic characteristic was the gender, as confirmed by the results and other researchers as well. Males are arguably more likely to choose the agricultural sector. It was also observed in our study that the unwillingness to work in agriculture would be higher among females. On the other hand, other studies confirmed that gender equality in agriculture was maintained in Lithuania; therefore, our study could be influenced by the fact that more females participated in the survey.

It should be noted that the present study had limitations, as it purposefully covered only the young people in the age group 15–19. Therefore, a more complex study could be carried out in the future. The further steps of the research will be the analysis of the youth's perceptions about different kinds of training and courses related to agricultural work. The present study has revealed that training has a positive effect on the willingness to work in agriculture. This may have been influenced by the fact that the youth participated in individual lessons about agriculture in schools.

## Data availability statement

The raw data supporting the conclusions of this article will be made available by the authors, without undue reservation.

## Ethics statement

Ethical review and approval was not required for the study on human participants in accordance with the local legislation and institutional requirements. Written informed consent from the participants was not required to participate in this study in accordance with the national legislation and the institutional requirements.

## Author contributions

LG, EB, AuN, and AnN contributed to preparation of methodology and collecting and analyzing the data. All authors contributed to the conceptualization, design, writing and editing of the article, read, reviewed, and approved the final paper and agreed to the published version of the manuscript.

## Funding

This article was developed under the work from European Cooperation in Science and Technology (COST) Action CA16123 called as SACURIMA—Safety Culture and Risk Management in Agriculture—https://www.sacurima.eu/.

## Conflict of interest

The authors declare that the research was conducted in the absence of any commercial or financial relationships that could be construed as a potential conflict of interest.

## Publisher's note

All claims expressed in this article are solely those of the authors and do not necessarily represent those of their affiliated organizations, or those of the publisher, the editors and the reviewers. Any product that may be evaluated in this article, or claim that may be made by its manufacturer, is not guaranteed or endorsed by the publisher.

## Footnotes

^1^World Bank Open Data. Access: https://data.worldbank.org/indicator/SL.AGR.EMPL.ZS?end=2019&most_recent_year_desc=false&start=1991&view=chart&year=2019.

^2^Eurostat database. Access: https://ec.europa.eu/eurostat/web/main/data/database.

^3^Eurostat database. Access: https://ec.europa.eu/eurostat/statistics-explained/index.php?title=Unemployment_statistics#Youth_unemployment.

^4^National Land Service under the Ministry of Agriculture of the Republic of Lithuania. Access: http://www.nzt.lt/go.php/lit/Lietuvos-respublikos-zemes-fondas.

^5^Employment Service Under the Ministry of Social Security and Labor of the Republic of Lithuania. Access: https://uzt.lt/wp-content/uploads/2019/07/%C5%BDem%C4%97s-%C5%ABkio-sektoriaus-tendencij%C5%B3-ap%C5%BEvalga-2019-06.pdf.

^6^Statistics Lithuania. Access: https://www.stat.gov.lt/home.

^7^Employment Service Under the Ministry of Social Security and Labor of the Republic of Lithuania. Access: https://uzt.lt/en/.

^8^Employment Service Under the Ministry of Social Security and Labor of the Republic of Lithuania. Access: https://osp.stat.gov.lt/darbo-rinka-lietuvoje-2020/uzimtumas-nedarbas-ir-laisvos-darbo-vietos/nedarbas.

^9^Civil Code of the Republic of Lithuania. Access: https://www.e-tar.lt/portal/lt/legalAct/TAR.8A39C83848CB/asr.

## References

[1] Cecchini M Bedini R Mosetti D Marino S Stasi S. Safety knowledge and changing behavior in agricultural workers: an assessment model applied in central Italy. Saf Health Work. (2018) 9:164–71. 10.1016/j.shaw.2017.07.009 29928530 PMC6005907

[2] Pfeifer GM. Pesticides, migrant farm workers, and corporate agriculture: how social work can promote environmental justice. J Progres Human Ser. (2016) 27:175–90. 10.1080/10428232.2016.1196428

[3] Kusis J Miltovica B Feldmane L. Latvian urban youth perceptions and stereotypes of farmer and agriculture. Econom Sci Rural Develop. (2014) 33:194–200.

[4] International Fund for Agricultural Development (IFAD). Creating opportunities for rural youth (2019). Rural Development Report. Available online at: https://www.ifad.org/documents/38714170/41190221/RDR2019_Overview_e_W.pdf/699560f2-d02e-16b8-4281-596d4c9be25a (accessed on 13 June 2021)

[5] Brinia V Papavasileiou P. Training of farmers in island agricultural areas: the case of cyclades prefecture. J Agricult Edu Exten. (2015) 21:235–47. 10.1080/1389224X.2014.928223

[6] Bello ARS Allajabou HA Baig MB. Attitudes of rural youth towards agriculture as an occupation: a case study from Sudan. Int J Develop Sustainabil. (2015) 4:415–24.

[7] Mueller V. Thurlow J. (2019). Youth and jobs in rural Africa: beyond stylized facts, 1st ed.; oxford university press: great clarendon street, Oxford, OX2 6DP, United Kingdom, 2019. Available online at: https://library.oapen.org/bitstream/handle/20.500.12657/37432/9780198848059.pdf?sequence=1 (accessed on 13 June 2021).

[8] Fawole WOB. Examining the willingness of youths to participate in agriculture to halt the rising rate of unemployment in South Western Nigeria. J Econom Stud. (2019) 46:578–90. 10.1108/JES-05-2017-0137

[9] Jansuwan P Zander KK. What to do with the farmland? Coping with ageing in rural Thailand. J Rural Stud. (2021) 81:37–46. 10.1016/j.jrurstud.2020.12.003

[10] Food and Agriculture organization of the United Nations (FAO). Youth and agriculture: Key challenges and concrete solutions. Published by the Food and Agriculture Organization of the United Nations (FAO) in collaboration with the Technical Centre for Agricultural and Rural Cooperation (CTA) and the International Fund for Agricultural Development (IFAD): Rome, (2014). Available online at: http://www.fao.org/3/i3947e/i3947e.pdf (accessed June 13, 2021).

[11] Mendez RG Pappalardo G Farrell B. Practicing fair and sustainable local food systems: elements of food citizenship in the simeto river valley. Agriculture. (2021) 11:56. 10.3390/agriculture11010056

[12] Pretty J. Agri-Culture: Reconnecting People, Land and Nature. London: Earthscan (2002).

[13] Food and Agriculture organization of the United Nations (FAO). The future of food and agriculture – Trends and challenges. Food and Agriculture Organization of the United Nations: Rome, (2017). Available online at: http://www.fao.org/3/i6583e/i6583e.pdf (accessed on 13 June 2021).

[14] Rigg J Phongsiri M Promphakping B Salamanca A Sripun M. Who will tend the farm? Interrogating the ageing Asian farmer. J Peasant Stud. (2020) 47:306–25. 10.1080/03066150.2019.1572605

[15] European Commission. Young farmers in the EU –structural and economic characteristics. EU Agricultural and Farm Economics Briefs. (2017) 15. Available online at: https://ec.europa.eu/info/sites/default/files/food-farming-fisheries/farming/documents/agri-farm-economics-brief-15_en.pdf (accessed June 13, 2021).

[16] Kołodziejczak W. Employment and gross value added in agriculture versus other sectors of the European Union Economy. Sustainability. (2020) 12:5518. 10.3390/su12145518

[17] Janeska V. Employment in agriculture and socio-economic development in the Republic of North Macedonia. Econom Develop. (2019) 1:9–23.

[18] Jöhr H. Where are the future farmers to grow our food? Int Food Agribus Manag Rev. (2012) 15:9–11.

[19] Halbeisen G Walther E Schneider M. Evaluative conditioning and the development of attitudes in early childhood. Child Dev. (2017) 88:1536–43. 10.1111/cdev.12657 27797098

[20] Vigoroso L Caffaro F Cavallo E. Occupational safety and visual communication: User-centred design of safety training material for migrant farmworkers in Italy. Saf Sci. (2020) 121:562–72. 10.1016/j.ssci.2018.10.029

[21] Dietrich C Buck E Specht A. Exploring the relationship between pre-schoolaged animated television and agriculture: a content analysis of Disney junior' s Mickey mouse clubhouse exploring the relationship between pre-school-aged animated television. J App Commun. (2015) 99:104–16. 10.4148/1051-0834.1065

[22] Kusis J Miltovica B Feldmane L. Lithuania and Latvia urban youth perceptions and stereotypes of farmer and agriculture. Reg Format Develop Stud. (2014) 14:148–56. 10.15181/rfds.v14i3.871

[23] Lundy LK Ruth AM Park TD. Entertainment and agriculture: an examination of the impact of entertainment media on perceptions of agriculture. J App Commun. (2007) 91:18. 10.4148/1051-0834.1257

[24] Volkov AA Morkunas M BaleŽentis T Ribašauskiene E Streimikiene D. A multi-criteria approach for assessing the economic resilience of agriculture: the case of lithuania. Sustainability. (2021) 13:2370. 10.3390/su13042370

[25] Morkunas M Labukas P. The evaluation of negative factors of direct payments under common agricultural policy from a viewpoint of sustainability of rural regions of the new EU member states: evidence from Lithuania. Agriculture. (2020) 10:228. 10.3390/agriculture10060228

[26] Volkov A Balezentis T Morkunas M Streimikiene D. Who benefits from CAP? The way the direct payments system impacts socioeconomic sustainability of small farms. Sustainability. (2019) 11:2112. 10.3390/su11072112

[27] Melnikiene R. Lietuvos Žemes ukio ir kaimo ekonomines, socialines ir aplinkosaugines situacijos vertinimas. Lithuanian Institute of Agrarian Economics. (2020). Available online at: https://zum.lrv.lt/uploads/zum/documents/files/BZUP_SP_SSGG_2020-10-30.pdf (accessed on 13 June 2021).

[28] Council Recommendation of 30 October 2020 on A Bridge to Jobs – Reinforcing the Youth Guarantee and replacing the Council Recommendation of 22 April 2013 on establishing a Youth Guarantee (2020/C 372/01). Available online at: https://eur-lex.europa.eu/legal-content/EN/TXT/HTML/?uri=CELEX:32020H1104(01)&from=EN (accessed on 13 June 2021).

[29] Magagula B Tsvakirai CZ. Youth perceptions of agriculture: influence of cognitive processes on participation in agripreneurship. Dev Pract. (2020) 30:234–43. 10.1080/09614524.2019.1670138

[30] Ahmad MI Oxley L Ma H. What makes farmers exit farming: a case study of Sindh Province, Pakistan. Sustainability. (2020) 12:3160. 10.3390/su12083160

[31] Utsugi T. (2012). Motivating factors for young adults in the brattleboro area to start in organic agriculture for their career. capstone collection, 2567. Available online at: https://digitalcollections.sit.edu/capstones/2567 (accessed May 15, 2021).

[32] European Commission,. Commission Staff Working Document: On EU Indicators in the Field of Youth. Brussels: European Commission (2021). Available online at: https://ec.europa.eu/assets/eac/youth/library/publications/indicator-dashboard_en.pdf (accessed June, 13 2021).

[33] Israel GD. Determining Sample Size. PEOD-6 (11), University of Florida: Florida Cooperative Extension Service. (1992). 1–5. Available online at: https://www.alnap.org/system/files/content/resource/files/main/pd00600.pdf (accessed May 15, 2021).

[34] Balezentis T Morkunas M Volkov A Ribasauskiene E Streimikiene D. Are women neglected in the EU agriculture? Evidence from Lithuanian young farmers. Land Use Policy. (2021) 101:105129. 10.1016/j.landusepol.2020.105129 33071421 PMC7547314

[35] Brauw De. Rural youth: determinants of migration throughout the world. IFAD: 2019 rural development report. (2019). Available online at: https://www.ifad.org/documents/38714170/41187395/15_de+Brauw_2019+RDR+BACKGROUND+PAPER.pdf/8a67f25f-749f-be91-f90f-beed3d524364 (accessed October 29, 2021).

[36] Ridha RN Wahyu BP. Entrepreneurship intention in agricultural sector of young generation in Indonesia. Asia Pacific J Innov Entrepren. (2017) 11:76–89. 10.1108/APJIE-04-2017-022

[37] Aziz A Naem N. Factors that influence the interest of youths in agricultural entrepreneurship. Int J Bus Soc Sci. (2013) 4:288–302.

[38] Simões F do Rio NB. How to increase rural NEETs professional involvement in agriculture? The roles of youth representations and vocational training packages improvement. J Rural Stud. (2020) 75:9–19. 10.1016/j.jrurstud.2020.02.007

[39] Baldwin C Smith T Jacobson C. Love of the land: social-ecological connectivity of rural landholders. J Rural Stud. (2017) 51:37–52. 10.1016/j.jrurstud.2017.01.012

[40] Liñán F Rodríguez-Cohard JC Rueda-Cantuche JM. Factors affecting entrepreneurial intention levels: a role for education. Int Entrep Manag J. (2011) 7, 195–218. 10.1007/s11365-010-0154-z

[41] Ismail LI Rabun MN Mohd Ali Nopiah AI. Are you inclined into agribusiness? Perspects Graduatg Stud J Int Bus Econom Entrepren. (2020) 5 30–38. 10.24191/jibe.v5i2.14233

[42] Akrong R Kotu BH. Economic analysis of youth participation in agripreneurship in Benin. Heliyon. (2022) 8:e08738. 10.1016/j.heliyon.2022.e08738 35071811 PMC8761699

[43] Vankov D Kozma D Galanternik M Chiers J Vankov B Wang L. Understanding the predictors of entrepreneurial intentions of young people from Argentina, Belgium, Bulgaria, China, and Romania. Entrepren Sustainabil Iss. (2022) 9:384–98. 10.9770/jesi.2022.9.3(23)

[44] Zhartay Z Khussainova Z Yessengeldin B. Development of the youth entrepreneurship: example of Kazakhstan. Entrepren Sustainabil Iss. (2020) 8:1190–208. 10.9770/jesi.2020.8.1(80)

[45] Paroda RS. Motivating and attracting youth in agriculture. Indian Journal. (2019) 8:149–56. 10.5958/2319-1198.2019.00011.3

[46] Nainggolan MF Nugraha DR Turnip A. Empowering of young farmer for arabica coffee farming business in Simalungun. In IOP Conf Series: Earth Environ Sci. (2020) 466:012034. 10.1088/1755-1315/466/1/012034

[47] Finger R Benni NE. Farm income in European agriculture: new perspectives on measurement and implications for policy evaluation. Eu Rev Agricul Econom. (2021) 48:253–65. 10.1093/erae/jbab011

[48] CFS policy recommendations on promoting youth engagement and employment in agriculture and food systems. zero draft – January (2022). Available online at: https://www.fao.org/fileadmin/templates/cfs/Docs2122/Youth/Zero_Draft/CFS_Policy_Recs_Youth_Zero_Draft.pdf (accessed July, 11 2022).

[49] Mujčinović A Nikolić A Tuna E Stamenkovska IJ Radović V Flynn P. Is it possible to tackle youth needs with agricultural and rural development policies? Sustainability. (2021) 13:8410. 10.3390/su13158410

